# Aramid Nanofiber/XNBR Nanocomposite with High Mechanical, Thermal, and Electrical Performance

**DOI:** 10.3390/nano13020335

**Published:** 2023-01-13

**Authors:** Jingyi Wang, Xumin Zhang, Yanwei Wen, Yang Chen, Quansheng Fu, Jing Wang, Hongbing Jia

**Affiliations:** 1 School of New Materials and Shoes & Clothing Engineering, Liming Vocational University, Quanzhou 362000, China; 2 Key Laboratory for Soft Chemistry and Functional Materials of Ministry of Education, Nanjing University of Science and Technology, Nanjing 210094, China; 3 Shanghai Institute of Aerospace Chemical Application, Huzhou 313002, China; 4 Professional Foundation Department, Changzhou Vocational Institute of Mechatronic Technology, Changzhou 213164, China

**Keywords:** nanocomposites, aramid nanofibers, polymer–matrix composites, multifunctional properties

## Abstract

Aramid nanofibers (ANFs) were successfully produced by deprotonation of Kevlar fiber followed by grafting epichlorohydrin in dimethyl sulfoxide solution. The ANFs were then incorporated into carboxylated acrylonitrile butadiene rubber (XNBR) by means of latex blending, followed by vulcanization. The interaction between ANFs and XNBR, and the effects of ANFs on the mechanical strength, dielectric properties, and thermal stability of ANF/XNBR nanocomposites were investigated. The results revealed that hydrogen bonding and covalent bonding interactions existed between ANFs and the XNBR matrix and played a critical role in the reinforcement of ANFs to XNBR nanocomposites. After adding 5 phr (parts per hundred rubber) of ANFs, the XNBR nanocomposite exhibited a significant improvement in mechanical properties, namely a 182% increase in tensile strength and a 101% increase in tear strength. In addition, the dielectric constant and thermal properties of ANF/XNBR also increased dramatically. ANFs may thus make an ideal candidate for high-performance rubber materials.

## 1. Introduction

Rubbers are of great industrial importance due to their high and reversible deformability. Fillers are one of the important ingredients in rubber composites as they improve the properties of rubber [1]. Recently, rubber nanocomposites with nanofillers have exhibited excellent mechanical properties as well as outstanding thermal properties compared with conventional rubber vulcanizates [2]. Aramid nanofibers (ANFs) are novel fibers with nanoscale dimensions that can possess a high tensile strength of 3.6 GPa and modulus of 90 GPa [3]. Through the deprotonation of amide groups of poly(para-phenylene terephthalamide) (PPTA) with potassium hydroxide (KOH) in the solution of dimethyl sulfoxide (DMSO), uniform ANFs are acquired. The ANFs exhibit a high aspect ratio at a diameter of 3–30 nm and a length of 5–10 μm [3]. It is considered that ANFs can be used as novel nanoscale building blocks for hybrid materials and generate a huge improvement in multiple properties of composites. For instance, Guan et al. [4] found that the incorporation of ANFs into poly(vinyl alcohol) (PVA) nanocomposites can enhance both the strength and toughness significantly due to the hydrogen bonds between PVA and ANFs. Wu et al. [5] fabricated hybrid membranes of combined ANFs and bacterial cellulose (BC), which possessed excellent transparency, flexibility, and mechanical strength. In our previous work [6], ANFs were utilized to prepare ANF/styrene–butadiene rubber (SBR) nanocomposites through latex co-coagulation. ANFs interacted with SBR via π–π stacking and generated a huge improvement in the performance of SBR nanocomposites.

Carboxylated acrylonitrile butadiene rubber (XNBR), which is composed of butadiene, acrylonitrile, and organic acid (acrylic acid, methacrylic acid, etc.), has widespread applications in the automobile, petroleum, mechanical engineering, and shipbuilding industries [7]. Nevertheless, the insufficient mechanical strength and heat resistance of pure XNBR restrict its qualification as a high-performance material. It has been reported that various nanoparticles, such as graphene oxide (GO) [7,8], multi-walled carbon nanotubes (MWCNTs) [9], and bacterial cellulose whiskers (BCWs) [10], can dramatically enhance the tensile strength and heat resistance of rubber nanocomposites. However, as far as we know, there have been few reports on the use of ANFs in XNBR.

In this work, ANFs were prepared through deprotonation of Kevlar fiber and then grafted with epichlorohydrin. An ANF aqueous suspension was incorporated into XNBR latex, followed by a latex co-coagulation and vulcanization to fabricate ANF/XNBR nanocomposites. The mechanical strength, dielectric properties, and thermal stability of ANF/XNBR nanocomposites were investigated.

## 2. Experience

### 2.1. Materials

XNBR latex (Nantex 6721, solid content: 43 wt.%) was bought from Zhenjiang Nantex Industry Co. Ltd., Zhenjiang, China. Kevlar 49 threads were bought from Dupont. Epichlorohydrin (ECH), potassium hydroxide (KOH), dimethyl sulfoxide (DMSO), and sodium chloride (NaCl) were supplied by Sinopharm Chemical Reagent Co., Ltd., Shanghai, China. Zinc oxide (ZnO), stearic acid (SA), N-cyclohexyl-2-benzothiazole sulfenamide (CZ), and sulfur (S) of industry grade were provided by Nanjing Jinsanli Rubber Plastic Co., Ltd., Nanjing, China.

### 2.2. Preparation of ANF/XNBR Nanocomposites

Deprotonation of Kevlar fibers was similar to previous reports [3,6]. Generally, Kevlar 49 yarns were snipped to debris and immersed in ethyl alcohol, with ultrasonic treatment for 6 h. Next, the aramid fibers were rinsed with deionized water and thoroughly dried. Then, 2.5 g aramid fibers and 4 g KOH were placed in 500 mL DMSO solution. The suspension was achieved after stirring for 7 days at 25 °C. After that, 2 mL ECH was instilled in the suspension and stirred at 30 °C for 1 day. The obtained suspension was centrifuged for 20 min, and the collected solids, named ANFs, were further cleaned with deionized water until the suspension was neutral. Finally, water-dispersible ANFs (5 mg/mL) were achieved by dispersing ANFs in deionized water with the help of ultrasonic treatment.

ANF/XNBR nanocomposites were prepared by latex co-coagulation of XNBR latex and ANF suspension. Typically, a certain amount of ANF suspension (5 mg/mL) was incorporated into XNBR latex and stirred for 2 h. After the co-coagulation with NaCl aqueous solution (6.5 wt.%), the rubber compound was cleaned with deionized water several times. The compound was dried in a vacuum oven at 60 °C until a fixed weight of compound was obtained. Afterward, rubber ingredients were mixed with the dried compound by an open two-roll mill. The basic recipe is shown as follows: XNBR 100.0 phr (phr, parts per hundred rubbers), SA 2.4 phr, ZnO 2.0 phr, CZ 2.2 phr, and S 1.5 phr, with ANFs variable. Finally, ANF/XNBR nanocomposites containing 0, 1, 3, 5, and 7 phr ANFs were achieved by compression molding at 160 °C and 15 MPa for the optimum curing time. The rubber nanocomposites were termed ANFs-x, where the x refers to ANFs’ loadings (phr) in nanocomposites.

### 2.3. Characterization

Fourier transform infrared spectroscopy (FTIR) was recorded on a FTIR-8400S spectrometer (Shimadzu Corporation, Kyoto, Japan) at a resolution of 4 cm^−1^. Ultraviolet-visible spectroscopy (UV-Vis) was carried out by a UV-6100S spectrophotometer (Shanghai Mapada Co., Ltd., Shanghai, China). Thermo-gravimetric analyses (TGA) measurement was carried out with a DTG-60 differential thermogravimetric (Shimadzu Co., Ltd., Japan) under an N_2_ atmosphere with a heating rate of 10 °C/min^−1^. X-ray diffraction (XRD) was measured by a D8-Advanced X-ray diffractometer (Bruker Corporation, Fällanden, Switzerland) with Cu Kα radiation (λ = 0.154 nm) at 25 °C. The samples were scanned from 10° to 30° at a scanning speed of 3°/min. The tensile strength and tear strength of samples were tested by a universal testing machine (Shenzhen SANS Co., Ltd., Shenzhen, China) at room temperature (25 °C) according to ASTM D-412 and ASTM D-624, respectively. At least five specimens of each sample were tested. The dynamic mechanical properties were tested using a dynamic mechanical analyzer (DMA1, METTLER TOLEDO, Stockholm, Sweden) at a heating rate of 3 °C/min from −60 °C to 40 °C and a tensile mode of 1.0 Hz. Dielectric performances were tested by a WK6550B precision impedance analyzer (Wayne Kerr, Bognor Regis, UK) with a frequency from 100 Hz to 5 MHz. The molecular dynamics (MD) simulations were performed with the Condensed-phase Optimized Molecular Potential for Atomistic Simulation Study (COMPASS) force field. In Materials Studio software, information about the hydrogen bond in the system was obtained through the toolbar “Calculate Hydrogen Bonds”, the hydrogen bond length set by the simulation was no more than 2.5 Å, and the angle was greater than 90°. After averaging the number of hydrogen bonds in the last 10 frames of each optimized cell structure, detailed information on hydrogen bonds in ANF/XNBR nanocomposites was obtained.

## 3. Results and Discussion

### 3.1. Interactions between XNBR and ANFs

**Figure 1:**
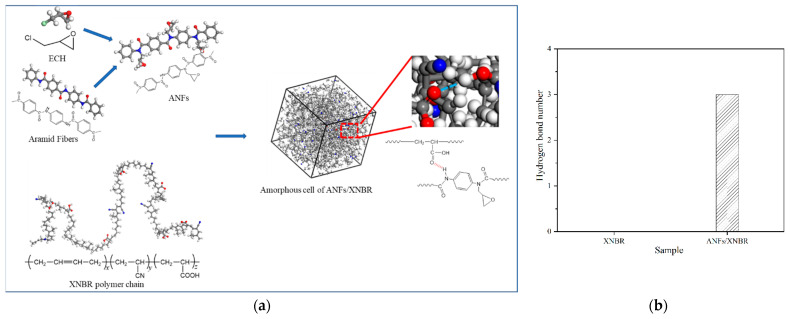
(**a**) Constructs of the nanocomposites models (the gray spheres are carbon atoms, the white spheres are hydrogen atoms, the blue spheres are nitrogen atoms, and the red spheres are oxygen atoms), and (**b**) the number of intermolecular hydrogen bonds.

The hydrogen bond is a strong intermolecular interaction and one of the important interactions of nanocomposites [11]. The formation of hydrogen bonds is conducive to the interface adhesion between filler and matrix. Therefore, MD simulations were performed, and the construction of the nanocomposite models is shown in Figure 1a. MD simulation can calculate the number and type of hydrogen bonds. In nanocomposites, the carboxyl group on XNBR is a good hydrogen bond donor/acceptor so it can form an intramolecular hydrogen bond [12]. There are three types of hydrogen bonds in ANF/XNBR composites (Figure S1). The first type of hydrogen bond is formed between ANFs and the XNBR molecular chains. The second type of hydrogen bond is formed between ANFs. The third kind of hydrogen bond is formed between XNBR molecular chains. Table S1 shows the types and number of hydrogen bonds in ANF/XNBR nanocomposites. However, these intramolecular hydrogen bonds have little effect on the interface interaction of composites. Thus, in this section, only the hydrogen bond between the filler and rubber molecular chain is discussed. The calculated result for the hydrogen bond between ANFs and XNBR is shown in Figure 1b. Compared with pure XNBR, the number of intermolecular hydrogen bonds between XNBR and ANFs increases, which means that there is a significant hydrogen bonding effect.

**Figure 2:**
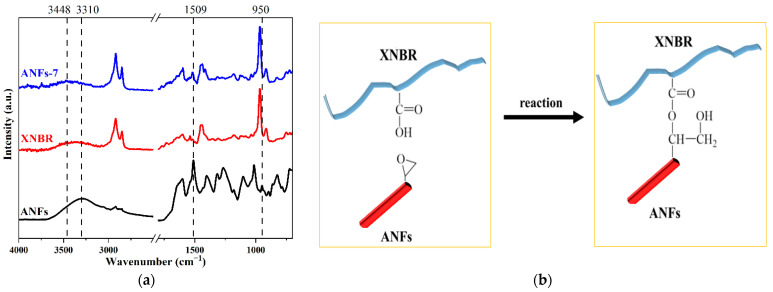
(**a**) FTIR spectra of ANFs and ANF/XNBR nanocomposites; (**b**) reaction between ANFs and XNBR.

In order to distinguish the interaction(s) between XNBR and ANFs, FTIR was used to detect the molecular bonds’ details. For neat XNBR (Figure 2), there is a wide and dull peak at 3448 cm^−1^ (O–H group stretching vibration of carboxyl groups). In the case of ANFs, compared with aramid fiber (Figure S2), the FTIR spectrum of ANFs shows a new peak at 950 cm^−1^ and a reduction in the characteristic peak at 1537 cm^−1^, which are ascribed to the vibration of the epoxy and amide groups. This means that the epoxy group of ECH has been grafted onto an aramid fiber. The FTIR spectrum of the ANFs-7 nanocomposite shows peaks at around 3310 cm^−1^ (N–H stretching vibration of ANFs) and 1509 cm^−1^ (C=C stretching vibrations of the aromatic ring of ANFs). In addition, it is observed that the peak at 950 cm^−1^ (epoxy group of ANFs) disappears in ANF/XNBR nanocomposites. This implies that the carboxyl groups of XNBR and epoxy groups of ANFs are involved in the formation of covalent bonds under high temperature and pressure conditions during the vulcanization process, as shown in Figure 2b. In previous work, Rocks et al. [13] studied the reaction between carboxyl and epoxy groups and a similar reaction mechanism was also noted. Therefore, XNBR and ANFs not only form effective hydrogen bonds but also form covalent bonds through carboxyl and hydroxyl groups.

**Figure 3:**
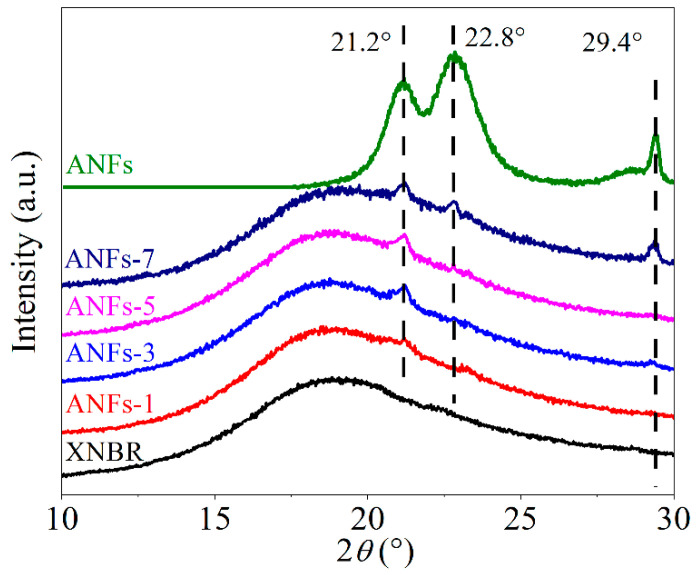
XRD patterns of ANFs and ANF/XNBR nanocomposites.

The XRD patterns of ANF/XNBR nanocomposites are shown in Figure 3. There is a single broad diffraction peak at 2θ = 18.7° for neat XNBR, which is assigned to the amorphous structure of XNBR [7]. For ANFs, typical diffraction peaks at around 21.2°, 22.8°, and 29.4° belong to (110), (200), and (004) lattice planes, respectively [3,14]. The XRD patterns of ANF/XNBR nanocomposites combine both the crystal feature of ANFs (peaks at 21.2°, 22.8°, and 29.4°) and the amorphous feature of XNBR (peak at 18.7°). Moreover, the intensity of characteristic peaks of ANFs (29.4°) in XNBR nanocomposites is increased with the addition of ANFs. These results demonstrate that ANFs are tightly embedded into the rubber matrix and preserve its crystal structure. In summary, this tight embedding of ANFs in the XNBR matrix and the interfacial interactions between ANFs and XNBR are critical to the improvement of the mechanical strength and thermal stability of nanocomposites, which are shown as follows.

### 3.2. Mechanical Properties

**Figure 4:**
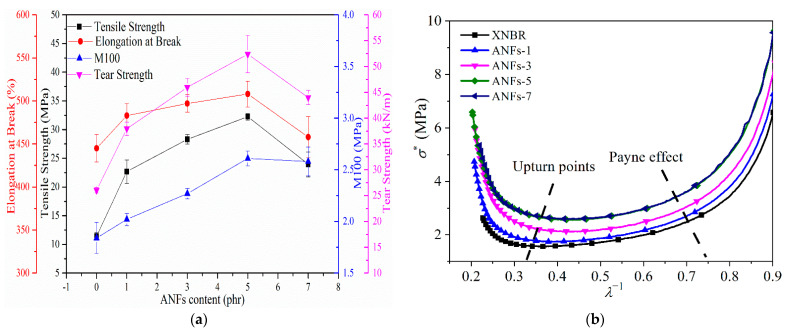
(**a**) Mechanical properties of ANF/XNBR nanocomposites; (**b**) Mooney–Rivlin plots of reduced stress as a function of the reciprocal extension ratio of nanocomposites.

The mechanical data for ANF/XNBR nanocomposites, including tensile strength, elongation at break, tensile modulus at 100% elongation (M100), and tear strength, are presented in Figure 4a. The tensile strength of ANF/XNBR nanocomposites increases with the addition of ANFs and achieves a maximum (32.26 MPa) with 5 phr ANFs, which is increased by 182% in contrast to neat XNBR. However, the tensile strength exhibits a decrease with higher ANF loading. The values of elongation at break, M100, and tear strength of ANF/XNBR nanocomposites show a similar tendency to the tensile strength, and the ANFs-5 nanocomposite has maximal increments of 14%, 42%, and 101% compared with those of the neat XNBR, respectively. The high strength and stiffness, as well as the high aspect ratio of ANFs, allow a high extent of stress sharing with the matrix [10], which is responsible for the excellent improvement in the mechanical performances of ANF/XNBR nanocomposites. The covalent bond and hydrogen bond interfacial interaction between ANFs and XNBR is another factor supporting the improved mechanical properties [15]. The rough fracture surface of rubber nanocomposites detected by SEM (Figure S3) also proves the enhanced mechanical properties. In the neat XNBR, there is a smooth fracture surface with little white dots (Figure S3a). With the incorporation of ANFs, there are many white dots on the rougher fracture surface of rubber nanocomposites (Figure S3b–d). These white dots may be the rigid ANFs embedded in the rubber matrix. The rougher fracture surface seems to have more energy dissipation during stretching, likely due to the higher value of strength. However, an excessive content of ANFs can result in agglomerations in the matrix and the stress transfer becomes blocked [16]. Consequently, this causes a decrease in the mechanical properties of nanocomposites.

Next, the rubber network was assessed by the classical Mooney–Rivlin equation, which is shown as follows [10]:

(1) $$\sigma^{*}\left(\lambda \right)=\frac{\sigma }{{\lambda -\lambda }^{-2}}{=2C}_{1}{+2C}_{2}\lambda^{-1}$$

where the σ* is the Mooney stress, *λ* is extension ratio, *σ* is nominal stress, and *C*_1_ and *C*_2_ are constants independent of *λ*. Figure 4b shows that the plots of ANF/XNBR nanocomposites exhibit a “U” form. In summary, the σ* of all filled rubber systems decreases sharply under low strain (*λ*^−1^ > 0.7). This is mainly due to the breakdown of the filler network and thus the release of the rubber trapped in the filler network, i.e., the Payne effect [17]. In addition, σ* of all filled rubbers shows a sudden rise in the range of high strain (*λ*^−1^ < 0.4). This can be attributed to the disentanglement of the XNBR macromolecule chains [10]. Furthermore, the value of σ* increases gradually with the increase in ANFs. For the ANF/XNBR nanocomposite, due to the reaction between the carboxyl group of XNBR and the hydroxyl group of ANFs, the ANFs play the role of a cross-linker in the nanocomposites, and the increase in ANFs causes more chemical cross-link points in the rubber matrix, which can enhance the rubber network.

### 3.3. Dynamic Mechanical Properties

**Figure 5:**
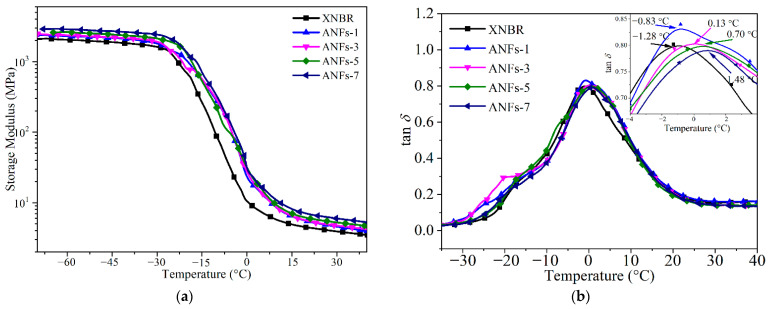
Temperature dependence of (**a**) *E*′ and (**b**) tan*δ* for ANF/XNBR nanocomposites.

To further investigate the reinforcement of ANFs on the rubber matrix, the dynamic mechanical properties of ANF/XNBR nanocomposites were estimated by DMA. Figure 5 represents the temperature dependence of the storage modulus (*E*′) and loss tangent (tan*δ*) for ANF/XNBR nanocomposites. Generally, *E*′ is in relation to the stiffness and load-bearing capacity of the materials [18]. The *E*′ of ANF/XNBR nanocomposites is enhanced with the increase in ANFs in both glassy and rubbery regions, suggesting that ANFs can improve the stiffness and load-bearing capacity of nanocomposites. This indicates the reinforcement of ANFs on the rubber matrix.

The glass transition temperature (*T*_g_) of ANF/XNBR nanocomposites can be decided by the peak of the tan*δ* vs. temperature plot, as presented in Figure 4b. The *T*_g_ of ANF/XNBR nanocomposites shifts to a higher temperature with the addition of ANFs (inset image), in contrast to that of neat XNBR. This is due to the low movement ability of rubber chain segments hindered by ANFs [17] and demonstrates the existence of strong interfacial interactions between ANFs and the XNBR matrix.

### 3.4. Dielectric Properties

**Figure 6:**
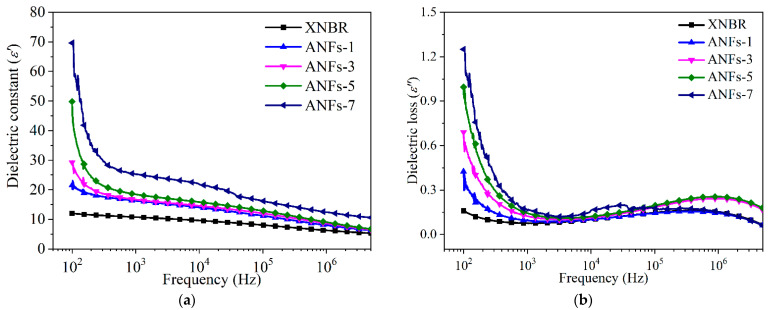
Dependence of (**a**) dielectric constants and (**b**) dielectric loss on the frequency of ANF/XNBR nanocomposites at room temperature.

Polymer dielectrics have widespread use in electromechanical systems, such as embedded capacitors with high charge storage capacity, where the high dielectric constant and low dielectric loss are needed [19,20]. Figure 6 illustrates the typical variation of complex permittivity of ANF/XNBR nanocomposites within a frequency range from 100 Hz to 5 MHz. It is well-known that the real part of complex permittivity (*ε*′) relates to the storage capability of electric energy and the imaginary part (*ε*″) represents the loss capability of electric energy [17]. It can be observed from Figure 6a that neat XNBR exhibits a negligible decrease in *ε*′ over the whole measured frequency, which is even considered as a constant. This poor dielectric response is mainly on account of the inadequate charge accumulation in the XNBR matrix [17]. However, for ANF/XNBR nanocomposites, the *ε*′ is elevated with the increase in ANFs, and the *ε*′ plots show that the typical frequency dispersion behaviors resulted from the relaxation process under the applied electric field [21]. For instance, at 1 kHz, *ε*′ increases from 10.58 of neat XNBR to 18.44 of ANFs-5 nanocomposite. The *ε*′ of nanocomposites attain high values under low frequencies, which is due to the dipole orientation polarization and multiple interfacial polarization (Maxwell–Wagner–Sillars effect) [22,23]. It is worth mentioning that interfacial polarization occurs in heterogeneous systems, which is due to the accumulation of free charges on the interface of the components [23]. Since the ANF/XNBR nanocomposite is a hierarchical structure, multi-interfaces emerge based on the uniform distribution of filler. There are more multi-interfaces within the system with the addition of more fillers. In addition, the high *ε*′ of nanocomposites at low frequencies also indicates the presence of electrode polarization, a parasitic effect originating from the formation of space charges on the interfaces between electrodes and the specimen [24]. With the increase in frequency, the dipoles in nanocomposites cannot orient themselves along the direction of the applied electric field, causing frequency dispersion behaviors and low *ε*′ at high frequencies [24].

Regarding the dielectric loss (Figure 6b), all nanocomposites filled with ANFs show downward trends with the increase in applied frequency, which is similar to the variation of the dielectric constant. It is worth noting that the *ε*″ of ANFs-5 nanocomposites is only about 0.14 at 1 kHz, which is lower than that of the reported XNBR nanocomposites with 0.5 vol.% GO, of which the *ε*″ is about 1.4 [25]. Hence, ANF/XNBR nanocomposites with outstanding dielectric performance (high dielectric constant and low dielectric loss) can be designed for applications in the capacitor fields.

### 3.5. Thermal Properties

**Figure 7:**
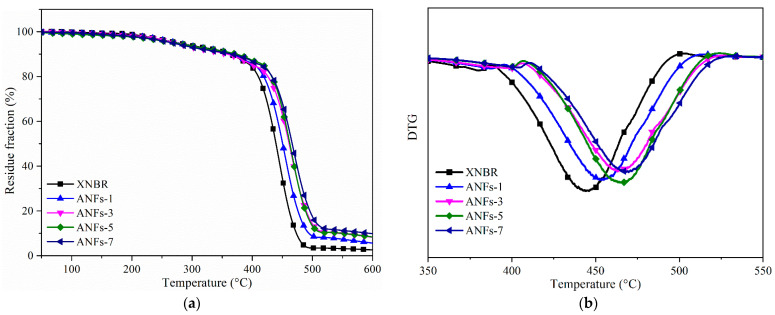
(**a**) TGA curves and (**b**) DTG curves of ANF/XNBR nanocomposites.

Thermogravimetric analysis was carried out to study the heat resistance of ANF/XNBR nanocomposites, and the plots of TGA and DTG are depicted in Figure 7. The data from TGA curves are presented in Table 1. The temperature for 5% degradation (*T*_5_) of nanocomposites decreases with the increase in ANFs, which is on account of the low decomposition temperature (320 °C) of ANFs. However, both the temperature for 50% degradation (*T*_50_) and the maximal rate of decomposition (*T*_max_) of nanocomposites increase continuously with the addition of ANFs. For example, the *T*_50_ and *T*_max_ of ANFs-5 increase by 23 °C and 22 °C, respectively, compared with those of neat XNBR. In our previous work [15], 4 phr GO improved the *T*_max_ of XNBR by only 11 °C. This is because ANFs exhibit great thermal stability with a *T*_max_ of 538 °C, which may bring about great improvement in the thermal stability of the XNBR matrix. Meanwhile, the rigid nanofibers form a stiff network in a matrix via covalent bond interactions, which can delay heat transport and lead to a higher decomposition temperature of nanocomposites [8].

## 4. Conclusions

ANFs were obtained by deprotonation of Kevlar, followed by grafting with ECH, and incorporated into XNBR nanocomposites. It was found that hydrogen bonding and covalent bonding interaction existed between ANFs and the XNBR matrix, which played a critical role in the enhancement of the XNBR matrix. The tensile strength and tear strength of ANFs-5 nanocomposites were increased by 182% and 101%, respectively, in contrast to those of neat XNBR. Meanwhile, the storage modulus, dielectric constant, and thermal properties of XNBR nanocomposites showed great improvement with the increase in ANFs.

## Figures and Tables

**Table 1:** TG parameters for ANF/XNBR nanocomposites.

Sample	*T*_5_/°C	*T*_50_/°C	*T*_max_/°C	*R*_600_/wt.%
XNBR	276	439	445	2.61
ANFs-1	274	450	453	5.54
ANFs-3	271	461	463	7.78
ANFs-5	270	462	467	7.90
ANFs-7	267	466	469	9.32

*R*_600_: residue weight at 600 °C.

## Data Availability

Correspondence and requests for materials should be addressed to H.J. (polymernjust@gmail.com).

## Supplementary Materials

The following supporting information can be downloaded at: https://www.mdpi.com/article/10.3390/nano13020335/s1, Figure S1: Three types of hydrogen bonds, (a) the first type of hydrogen bond (type 1), (b), the second type of hydrogen bond (type 2), (c) the third type of hydrogen bond (type 3). (The gray spheres are carbon atoms, the white spheres are hydrogen atoms, the blue spheres are nitrogen atoms, the red spheres are oxygen atoms, and blue dashed line represents hydrogen bond); Figure S2: FTIR spectra of Aramid Fiber and ANFs; Figure S3: SEM images of ANF/XNBR nanocomposites (a) XNBR, (b) ANFs-1, (c) ANFs-3, (d) ANFs-5; Table S1: The number of hydrogen bonds of ANFs and ANF/XNBR.
