# Supplementary material for: Aramid Nanofiber/XNBR Nanocomposite with High Mechanical, Thermal, and Electrical Performance
Source: Nanomaterials (Basel). 2023 Jan 13;13(2):335. doi: 10.3390/nano13020335 (PMC9860882; doi:10.3390/nano13020335)
Supplement: Supplementary file 1 [file nanomaterials-13-00335-s001.zip › nanomaterials-2070696-supplementary.pdf]

Supporting Information

# Aramid Nanofiber/XNBR Nanocomposite with High Mechanical, Thermal, and Electrical Performance

Jingyi Wang[1, 2, †], Xumin Zhang[2, †], Yanwei Wen[2, 3], Yang Chen[2], Quansheng Fu[2], Jing Wang[4], Hongbing Jia[2*]

1 School of New Materials and Shoes & Clothing Engineering, Liming Vocational University, Quanzhou, 362000, China

2 Key Laboratory for Soft Chemistry and Functional Materials of Ministry of Education, Nanjing University of Science and Technology, Nanjing 210094, China

3 Shanghai Institute of Aerospace Chemical Application, Huzhou 313002, China

4 Professional Foundation Department, Changzhou Vocational Institute of Mechatronic Technology, Changzhou, Jiangsu, 213164, China

† These authors contributed equally to this work.

* Correspondence: polymernjust@gmail.com (H. Jia)

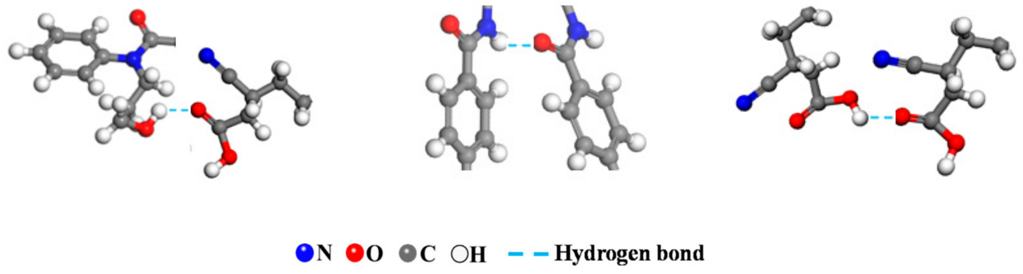


(a) (b) (c)

**Figure S1** Three types of hydrogen bonds, (a) the first type of hydrogen bond (type 1), (b), the second type of hydrogen bond (type 2), (c) the third type of hydrogen bond (type 3). (The gray spheres are carbon atoms, the white spheres are hydrogen atoms, the blue spheres are nitrogen atoms, the red spheres are oxygen atoms, and blue dashed line represents hydrogen bond)

**Table S1** The number of hydrogen bonds of ANFs and ANF/XNBR

| Samples | type 1 | type 2 | type 3 |
|---|---|---|---|
| XNBR | 0 | 0 | 5 |
| ANF/XNBR | 3 | 1 | 2 |

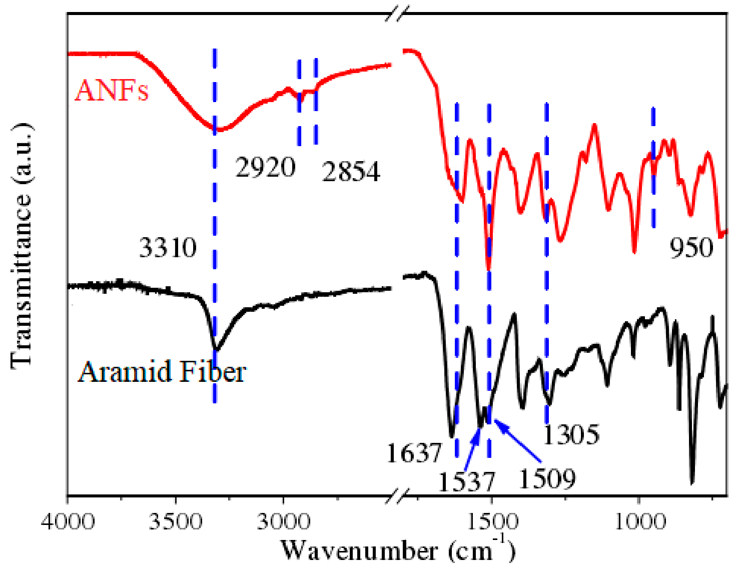


**Figure** S2 FTIR spectra of Aramid Fiber and ANFs

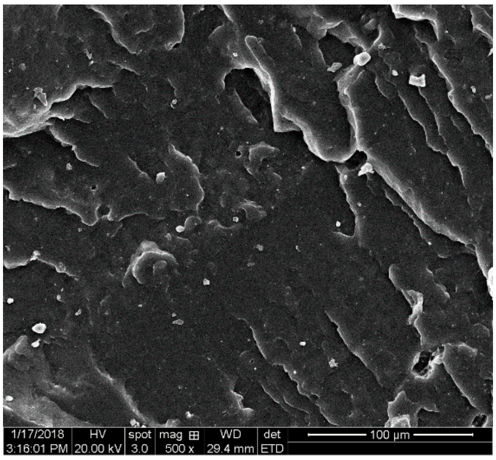


(a)

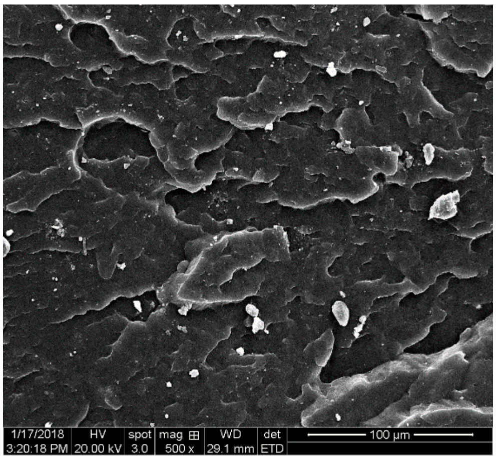


(b)

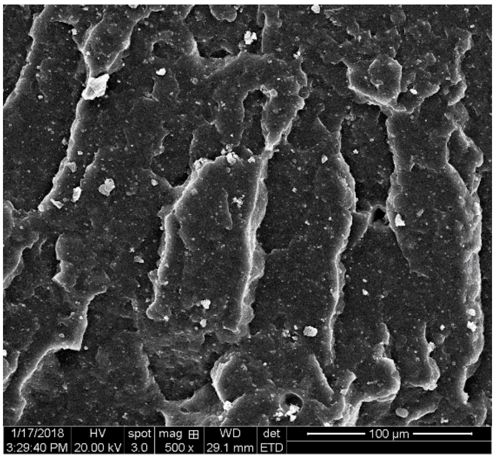


(c)

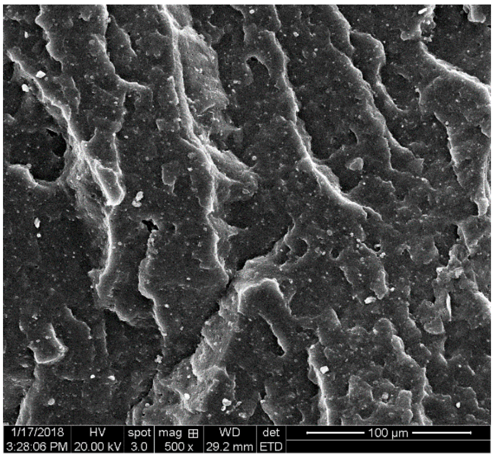


(d)

**Figure** S3. SEM images of ANF/XNBR nanocomposites (a) XNBR, (b) ANFs-1, (c) ANFs-3, (d) ANFs-5.
